# *De novo* assembly and transcriptome characterization: novel insights into catechins biosynthesis in *Camellia sinensis*

**DOI:** 10.1186/s12870-014-0277-4

**Published:** 2014-10-15

**Authors:** Zhi-Jun Wu, Xing-Hui Li, Zhi-Wei Liu, Zhi-Sheng Xu, Jing Zhuang

**Affiliations:** Tea Science Research Institute, College of Horticulture, Nanjing Agricultural University, Nanjing, 210095 China; State Key Laboratory of Crop Genetics and Germplasm Enhancement, College of Horticulture, Nanjing Agricultural University, Nanjing, 210095 China

**Keywords:** *Camellia sinensis*, Transcriptome, High-throughput sequencing, Catechins, RP-HPLC, Genetic diversity

## Abstract

**Background:**

Tea is a popular natural non-alcoholic beverage consumed worldwide due to its bioactive ingredients, particularly catechins (flavan-3-ols). Catechins not only contribute to tea quality but also serve important functions in the anti-stress regulation of secondary metabolic pathways. However, the percentages of various catechins are different among tea plant [*Camellia sinensis* (L.) O. Kuntze] cultivars. This study aimed to elucidate the biosynthetic mechanism of catechins. Transcriptomes from leaf tissues of four tea plant cultivars, ‘Yunnanshilixiang’, ‘Chawansanhao’, ‘Ruchengmaoyecha’, and ‘Anjibaicha’, were sequenced using the high-throughput sequencing platform Illumina HiSeq^™^ 2000. *De novo* assemble were also performed. Catechins contents were measured through reversed-phase high-performance liquid chromatography (RP-HPLC), and the biosynthetic pathway was also surveyed.

**Results:**

We constructed a unified unigene database. A total of 146,342 pairs of putative orthologs from the four tea plant cultivars, ‘Yunnanshilixiang’, ‘Chawansanhao’, ‘Ruchengmaoyecha’, and ‘Anjibaicha’ were generated. Approximately 68,890 unigenes (47.1%) were aligned to the sequences of seven public databases with a cut-off *E*-value of 1E-5. A total of 217 differentially expressed genes were found through RPKM values, and 150 unigenes were assigned to the flavonoid biosynthetic pathway using the integrated function annotation. The (−)-EGC and (−)-EC contents were significantly lower and the (+)-GC and (+)-C contents were abnormally higher in ‘Ruchengmaoyecha’ than in ‘Yunnanshilixiang’, ‘Chawansanhao’, and ‘Anjibaicha’. The proportion of catechins was confirmed by selecting critical genes (*ANS*, *ANR*, and *LAR*) for qRT-PCR analysis.

**Conclusions:**

This study provided a global survey of transcriptomes from four tea plant cultivars and serves as an available resource of genetic diversity. The analyses of transcriptome profiles and physiological indicators not only identified the putative genes involved in the flavonoid biosynthetic pathway but also provided some novel insights for the mechanisms of catechins biosynthesis.

**Electronic supplementary material:**

The online version of this article (doi:10.1186/s12870-014-0277-4) contains supplementary material, which is available to authorized users.

## Background

The tea plant, *Camellia sinensis* (L.) O. Kuntze, is naturally distributed in the Southeast Asia Monsoon region and has been cultivated in China as a commercially valuable plant for at least 2000 years [1,2]. Tea is made from tea plant leaves and is consumed as a popular natural non-alcoholic beverage worldwide due to its bioactive ingredients, including tea polyphenols [3], theanine [4], and polysaccharides [5]. Numerous reports revealed that tea prevents cancer, cardiovascular, neurodegenerative, and other oxidative stress-related diseases [6-10]. Green tea, black tea or tea constituents have been shown to inhibit the development of cancer in animal models, such as lung tumorigenesis in A/J mice [11,12] and intestinal tumorigenesis in *Apc*^min/+^ mice [13]. Population studies suggested that green and black tea consumption could reduce the risk for cardiovascular disease [14]. The potent antioxidant and iron chelating actions of tea extracts were shown to attenuate the neurotoxic action of 6-hydroxydopamine (6-OHDA)-induced neuronal death [7]. Moreover, tea also has been shown to prevent skin aging, liver cell injury and inflammation [15-17]. The benefits of tea are mainly attributed to catechins (flavan-3-ols), a group of polyphenolic compounds [18]. As tea principal flavor substances, catechins usually account for 25% to 30% of the dry weight of fresh tea plant leaves [19,20]. The accumulation of catechins in shoots may be related to energy storage and stress resistance [21-23].

The catechins in fresh tea leaves are usually classified into seven groups: (+)-gallocatechin [(+)-GC], (−)-epigallocatechin [(−)-EGC], (−)-epicatechin [(−)-EC], (+)-catechin [(+)-C], (−)-epigallocatechin gallate [(−)-EGCG], (+)-gallocatechin gallate [(+)-GCG], and (−)-epicatechingallate [(−)-ECG]. The flavonoid biosynthetic pathway of *C. sinensis* has been identified by numerous physiological, biochemical, and genetic studies [24-27]. However, the molecular mechanisms of (−)-EGCG, (+)-GCG, and (−)-ECG remain unclear to date. Catechins such as (−)-EGC, (−)-EC, (+)-GC, and (+)-C are synthesized through the enzymatic catalysis of anthocyanidin synthase (ANS), leucoanthocyanidin reductase (LAR), and anthocyanidin reductase (ANR) in the late stage of flavonoid biosynthesis [24]. (−)-EGCG and (−)-ECG may be biosynthesized by a newly discovered enzyme (epicatechin:1-*O*-galloyl-*β*-D-glucose *O*-galloyltransferase) [27]. The genes that encode these enzymes have been cloned or verified from *C. sinensis*, but information on their regulatory mechanisms remains lacking. The tea plant has a large genome [28,29]. Compared to other sequenced model plants, the genome size of tea plant (a perennial woody plant, ~4,000 Mb) is about 32.0, 9.3, 8.4, 8.2 times than that of two annual herbaceous model plants, *Arabidopsis thaliana* (125 Mb) [30] and rice (*Oryza sativa*, 430 Mb) [31,32], and two perennial woody model plants, grapevine (*Vitis vinifera*, 487 Mb) [33,34] and black cottonwood poplar (*Populus trichocarpa*, 485 Mb) [35]. Some genes are involved in flavonoid biosynthesis, and almost all genes may have multiple copies. Only a few flavonoid biosynthetic genes in *C. sinensis* have been completely cloned and functionally identified. The catechins content of different tea plant cultivars are different from one another. However, the exact mechanism responsible for this difference remains unclear.

**Figure 1:**
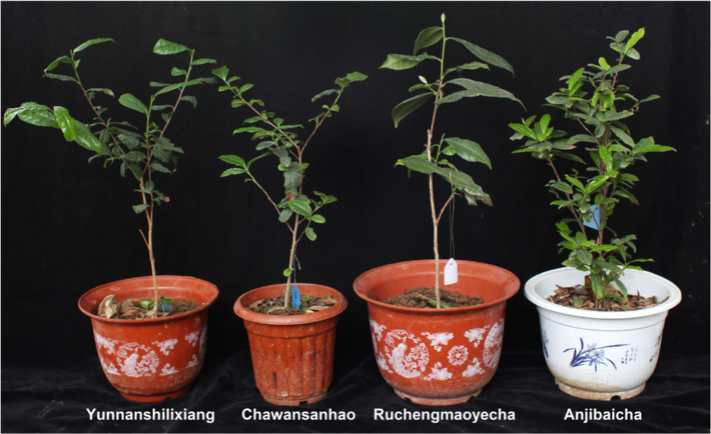
**Four tea plant cultivars: ‘Yunnanshilixiang’, ‘Chawansanhao’, ‘Ruchengmaoyecha’, and ‘Anjibaicha’.**

This study elucidated the mechanisms and critical genes that regulate catechins biosynthesis. Transcriptomes of four tea plant cultivars from different provinces in China were sequenced using the high-throughput sequencing platform Illumina HiSeq™ 2000 and were *de novo* assembled. The tea plant samples used here included mid-leaf ‘Yunnanshilixiang’ (Tea_T1) from Yunnan province, small-leaf ‘Chawansanhao’ (Tea_T2) from Jiangsu province, high-temperature-tolerant large-leaf ‘Ruchengmaoyecha’ (Tea_T3) from Hunan province, and low-temperature-sensitive small-leaf ‘Anjibaicha’ (Tea_T4) from Zhejiang province (Figure 1). Because of the obvious difference of geographic and climate characteristics in these four tea production areas, respectively plateau monsoon climate (Yunnan), coastal temperate climate (Jiangsu), inland subtropical monsoon climate (Hunan), and coastal subtropical monsoon climate (Zhejiang), the morphology and physiology of tea plants of Tea_T1, Tea_T2, Tea_T3, and Tea_T4 were different, such as leaf size and environmental adaptability. The contents and component proportions of catechins compounds are one of the important factors of the characteristics of tea-processing suitability and quality [36,37]. The tea plant cultivars of ‘Yunnanshilixiang’, ‘Chawansanhao’, and ‘Anjibaicha’ are suitable processed into green tea, however, ‘Ruchengmaoyecha’ is suitable for black tea.

The same set of reference genes was established to analyze the subsequent expression abundance and the reads per kb per million reads (RPKM) values of two libraries. A total of 217 differentially expressed unigenes were identified. Function annotation analysis showed that 150 unigenes are involved in flavonoid biosynthetic pathway. Seven genes (*LAR-1*, *LAR-2*, *LAR-3*, *ANS-1*, *ANS-2*, *ANR-1*, and *ANR-2*) that encode for three key enzymes (ANS, ANR, and LAR) had different expression patterns among the four tea plant cultivars, in which expression of six genes (*LAR-2*, *LAR-3*, *ANS-1*, *ANS-2*, *ANR-1*, and *ANR-2*) positively associated with the concentration of their corresponding catechins. Another gene *LRA-1* may not be primarily responsible for the biosynthesis of catechins, replaced by the same set of genes *LRA-2* and *LRA-3*. Our study may served as a reference for further studies on the multi-gene regulation of catechins biosynthesis in *C. sinensis*.

## Results

### Sequencing and *de novo* assembly

Four cDNA libraries were constructed from fresh leaves RNA samples of Tea_T1, Tea_T2, Tea_T3, and Tea_T4. Approximately 25.7, 21.5, 20.7, and 27.1 million raw reads of 200 bp, and 5.1, 4.3, 4.2, and 5.4 Giga base pairs (Gbp) each, respectively, were generated by the Illumina HiSeq™ 2000 sequencing device. The Q20 values (sequencing error rate, 1%) were more than 93.75%, and the GC percentages were 44.41%, 46.65%, 51.08%, and 46.09%, respectively (Table 1). Adaptor sequences, duplicated sequences, ambiguous reads, and low-quality reads were removed, and the high-quality reads of each cultivar were separately *de novo* assembled using the Trinity program [38]. The assembly finally produced 86,523 unigenes with the mean size of 591 bp for Tea_T1, 54,980 unigenes with the mean size of 601 bp for Tea_T2, 34,442 unigenes with the mean size of 530 bp for Tea_T3, and 74,894 unigenes with the mean size of 596 bp for Tea_T4 (Table 1).

**Table 1:** **Summary of the sequence assembly for four cultivars of** ***C. sinensis***

	**Species**	**Assembly size (n)**	**Nucleotides (bp)**	**GC%**	**Q20%**	**Average length (bp)**	**N50 (bp)**
Raw reads	Tea_T1	25,733,467	5,118,861,578	44.41	95.70	-	-
	Tea_T2	21,524,046	4,294,394,519	46.65	95.08	-	-
	Tea_T3	20,674,230	4,163,349,704	51.08	93.75	-	-
	Tea_T4	27,082,850	5,387,399,257	46.09	94.21	-	-
Unigene reads	Tea_T1	86,523	51,143,990	-	-	591	829
	Tea_T2	54,980	33,028,924	-	-	601	923
	Tea_T3	34,442	18,248,161	-	-	530	700
	Tea_T4	74,894	44,645,039	-	-	596	890

**Figure 2:**
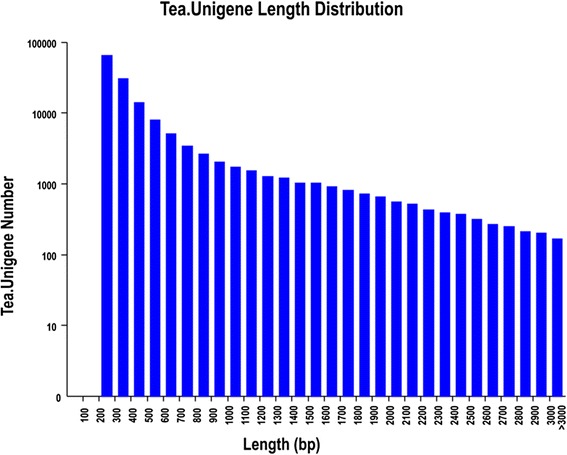
**Length distribution of the Tea.Unigene library of** ***C. sinensis*** **.**

The same set of reference genes (Tea.Unigene library) was analyzed to determine the subsequent expression abundance and differentially expressed genes. A total of 146,342 unigenes were obtained from the tea plant cultivars, with an average unigenes and N50 length were 526 bp and 648 bp, respectively (Table 2). The length distribution of the unigenes is shown in Figure 2.

**Table 2:** **Statistics of the Tea.Unigene library of** ***C. sinensis***

**Tea.Unigene length**	**Total number**	**Percentage**
200-300	64,917	44.36%
300-500	44,409	30.35%
500-1000	21,098	14.42%
1000-2000	10,812	7.39%
2000+	5,106	3.49%
Total number	146,342	
Total length	76,924,597	
N50 length	648	
Mean length	526

### Functional annotation and categorization

All unique sequences were annotated using BLASTX against the NCBI non-nucleotide (Nt) sequences database, NCBI non-redundant (Nr) protein database, Gene Ontology (GO), Kyoto Encyclopedia of Genes and Genomes (KEGG) [39], Clusters of Orthologous Groups (COG) [40], and UniProtKB/(Swiss-Prot, TrEMBL) [41] to annotate the assembly as comprehensively as possible. A total of 68,890 unigenes (47.07%) were identified with a significance threshold (*E*-value ≤1E-5). The remaining unigenes (52.93%) cannot be annotated with known genes (Table 3), which most likely caused by the presence of short sequences (44.36% <300) and the shortage of relevant genetic data.

**Table 3:** **Summary of annotation for the Tea.Unigene library of** ***C. sinensis***

**Anno database**	**Annotated number**	**300 ≤ length < 1000**	**length ≥ 1000**
COG annotation	17,028	7,048	6,398
GO annotation	50,846	23,494	14,047
KEGG annotation	15,300	7,113	3,898
Swissprot annotation	44,936	20,674	13,214
TrEMBL annotation	58,446	27,648	15,137
Nr annotation	58,678	27,829	15,152
Nt annotation	45,838	19,557	13,829
All annotated	68,890	32,770	15,348

**Figure 3:**
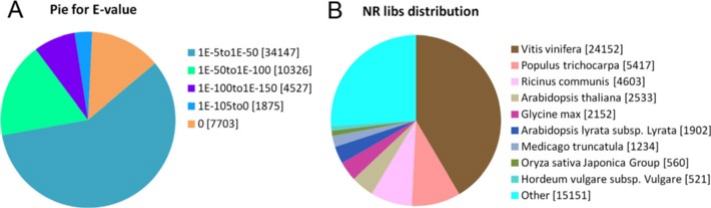
**Characteristics of the homology search of the Tea.Unigene library of** ***C. sinensis*** **against the nr database. (A)** *E*-value distribution of BLASTX hits for each unigene with a cut-off of 1E-5. **(B)** Species distribution of the top nine BLAST hits for each unigene with a cut-off of 1E-5.

*E*-value and species distribution were also analyzed by evaluating the matched unigenes (58,678) from the returned BLASTX results of the Nr protein database. Very strong homology was observed in 41.71% of the aligned sequences (*E* <1E-50), and 58.29% of the homolog sequences ranged from 1E-50 to 1E-5 (Figure 3A). The species distribution of the top hits that matched the sequences showed that *Vitis vinifera* (41.16%) had the greatest number of matches with *C. sinensis*, followed by *Populus trichocarpa* (9.23%), *Ricinus communis* (7.84%), *Arabidopsis thaliana* (4.32%), *Glycine max* (3.67%), *Arabidopsis lyrata* (3.25%), *Medicago truncatula* (2.10%), *Oryza sativa* Japonica Group (0.95%) and *Hordeum vulgare* (0.88%) (Figure 3B).

### GO classification

**Figure 4:**
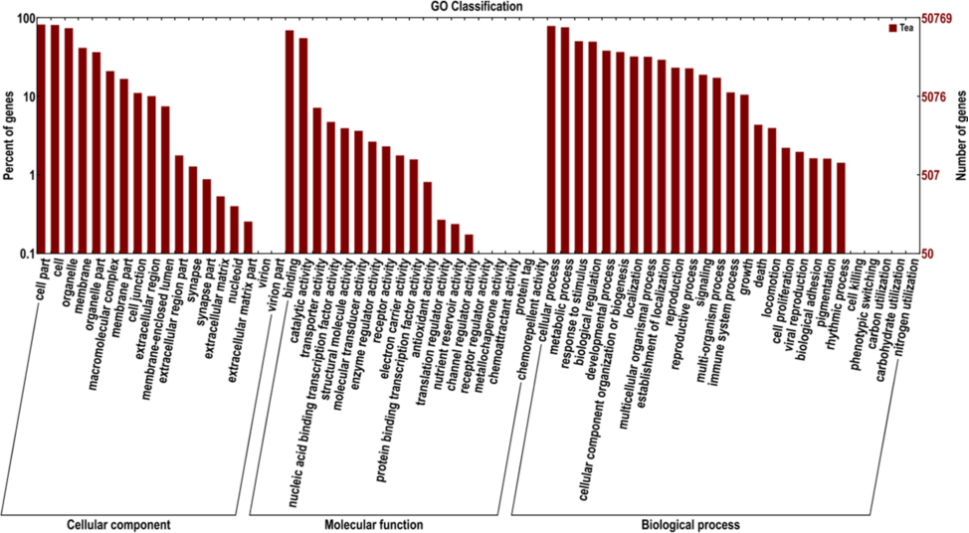
**GO classification of the Tea.Unigene library of** ***C. sinensis*** **.**

The expressed *C. sinensis* genes were searched against the GO database to categorize standardized gene functions. Of the 58,678 unigenes previously annotated to the NR database, 50,846 were assigned to three main GO categories (biological process, cellular component, and molecular function) and 64 subcategories using the Blast2GO and WEGO software (Figure 4). A total of 14,278 GO terms were collected, which were most frequently related to biological processes (9,106), followed by molecular function (3,843), and cellular components (1,329).

The major subcategories (above 25% genes) among the biological processes were “cellular process” (78.74%, 40,036), “metabolic process” (75.78%, 38,532), “response to stimuli” (50.38%, 25,615), “biological regulation” (49.66%, 25,248), “developmental process” (38.08%, 19,362), “cellular component organization or biogenesis” (36.68%, 18,649), “localization” (32.11%, 16,326), “multicellular organismal process” (31.90%, 16,219), “establishment of localization” (29.18%, 14,838), “reproduction” (23.12%, 11,756), and “reproductive process” (22.73%, 11,556). “Binding” (69.38%, 35,276) and “catalytic activity” (55.20%, 28,068) were the dominant molecular functions. The most highly represented cellular component was “cell part” (82.15%, 41,770), followed by “cell” (81.26%, 41,320), “organelle” (73.86%, 37,553), “membrane” (41.36%, 21,031), “organelle part” (36.49%, 18,553), and “macromolecular complex” (21.02%, 10,686).

### COG classification

**Figure 5:**
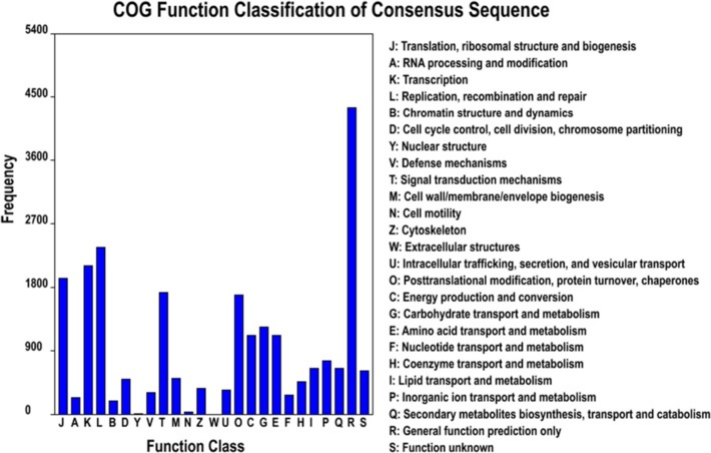
**COG function classification of the Tea.Unigene library of** ***C. sinensis*** **.**

COG was used to further evaluate the completeness of the tea plant transcriptome libraries and the validity of the annotation. A total of 17,028 unigenes were clustered into 25 functional categories (Table 3). The largest category was “General function prediction only” (25.52%, 4,345), followed by “replication, recombination and repair” (13.88%, 2,363), “transcription” (12.38%, 2,108), “translation, ribosomal structure and biogenesis” (11.35%, 1,933), “Signal transduction mechanisms” (10.14%, 1,726), “Posttranslational modification, protein turnover, chaperones” (9.95%, 1,695), “Carbohydrate transport and metabolism” (7.32%, 1,246), “Energy production and metabolism” (6.62%, 1,127), and “Amino acid transport and metabolism” (6.60%, 1,124). “Extracellular structure”, “nuclear structure”, and “cell motility” had the fewest unigenes (Figure 5).

### KEGG classification

**Figure 6:**
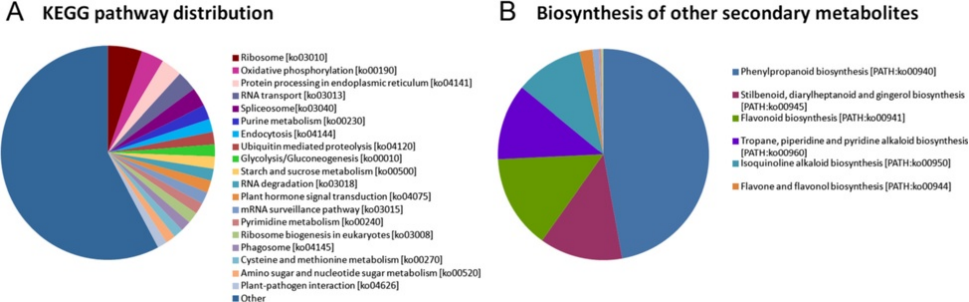
**KEGG classification of the Tea.Unigene library of** ***C. sinensis*** **. (A)** KEGG pathway distribution of the Tea.Unigene library of *C. sinensis*. **(B)** KEGG pathway involved in the biosynthesis of other secondary metabolites.

To further explore the gene interactions and biological functions in *C. sinensis* leaves, the unigenes were searched against the reference canonical pathways in KEGG. A total of 15,300 unigenes were annotated with their corresponding Enzyme Commission (EC) numbers and were assigned to 251 KEGG pathways (Table 3, see Additional file 1). The most representative pathways were “ribosome” (ko03010, 856, 5.59%), “oxidative phosphorylation” (ko00190, 564, 3.69%), “protein processing in endoplasmic reticulum” (ko04141, 522, 3.41%), “RNA transport” (ko03013, 507, 3.31%), “spliceosome” (ko03040, 473, 3.09%), “purine metabolism” (ko00230, 355, 2.32%), “endocytosis” (ko04144, 325, 2.12%), “ubiquitin-mediated proteolysis” (ko04120, 311, 2.03%), “glycolysis/gluconeogenesis” (ko00010, 308, 2.01%), “starch and sucrose metabolism” (ko00500, 307, 2.01%), “RNA degradation” (ko03018, 299, 1.95%), “plant hormone signal transduction” (ko04075, 299, 1.95%), “mRNA surveillance pathway” (ko03015, 293, 1.92%), “pyrimidine metabolism” (ko00240, 282, 1.84%), “ribosome biogenesis in eukaryotes” (ko03008, 280, 1.83%), “phagosome” (ko04145, 259, 1.69%), and “cysteine and methionine metabolism “(ko00270, 244, 1.59%) (Figure 6A).

This study focused on the “Biosynthesis of other secondary metabolites” pathway present in *C. sinensis* leaves and revealed 140 unigenes for “Phenylpropanoid biosynthesis”, 38 unigenes for “Stilbenoid, diarylheptanoid, and gingerol biosynthesis”, 43 unigenes for “Flavonoid biosynthesis”, 35 unigenes for “Tropane, piperidine, and pyridine alkaloid biosynthesis”, 31 unigenes for “Isoquinoline alkaloid biosynthesis”, 6 unigenes for “Flavone and flavonol biosynthesis”, 3 unigenes for “Novobiocin biosynthesis”, 2 unigenes for “Caffeine metabolism”, 1 unigene for “Indole alkaloid biosynthesis”, and 1 unigene for “Streptomycin biosynthesis” (Figure 6B).

### Differential gene expression in the tea plant libraries

**Figure 7:**
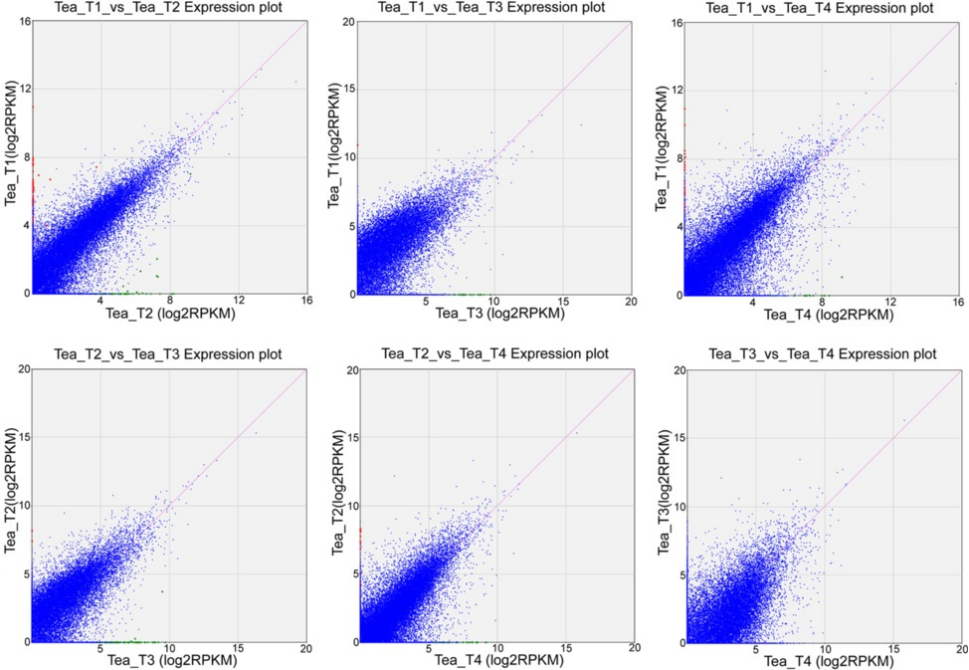
**Distribution of the differentially expressed genes between two** ***C. sinensis*** **cultivars.**

**Figure 8:**
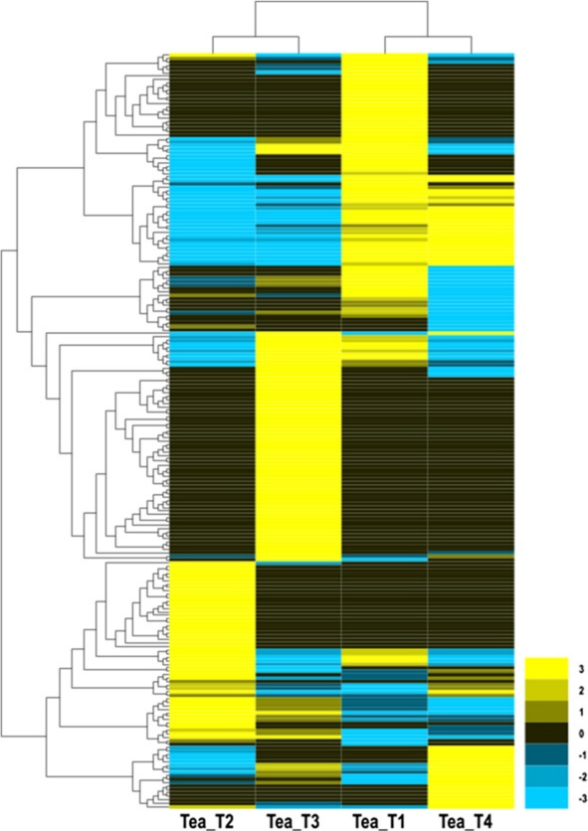
**Heatmap of the relative expression levels of 217 differentially expressed genes from four** ***C. sinensis*** **cultivars.** Yellow represents high expression. Blue represents low expression.

The expression abundance of each sample was measured, and differentially expressed genes (DEGs) were found between the two libraries. Clean reads from each sample were mapped back to the above-constructed reference genes, and the mapped reads were counted to obtain RPKM values for evaluation. A total of 273 DEGs were detected among the four *C. sinensis* libraries, of which 106, 25, 39, 64, and 39 DEGs were predicted from “Tea_T1_vs_Tea_T2”, “Tea_T1_vs_Tea_T3”, “Tea_T1_vs_Tea_T4”, “Tea_T2_vs_Tea_T3”, and “Tea_T2_vs_Tea_T4”, respectively, no DEGs were found in “Tea_T3_vs_Tea_T4” (Figure 7). Overlapping genes were removed, and 217 DEGs were obtained and hierarchically clustered. The gene expression profiles are shown in a heat map (Figure 8). The enriched genetic annotation for DEGs was analyzed, and the COG, GO, KEGG, Swissprot, TrEMBL, Nr, and Nt databases were annotated to describe the functions and metabolism of the genes compared with the transcriptome database (P ≤0.05, hypergeometric test). The detailed results are given in Additional file 2.

### Genes involved in the flavonoid biosynthetic pathway based on tea plant leaf transcriptome

**Figure 9:**
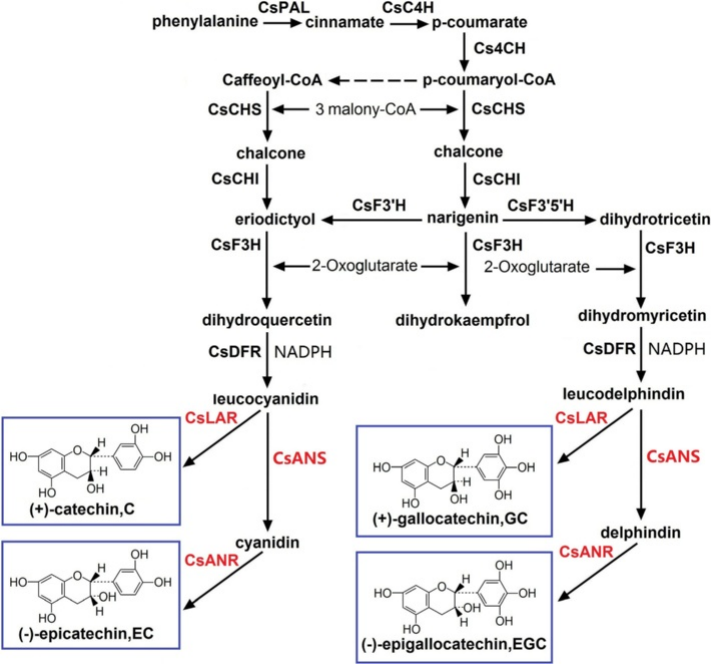
**Unigenes involved in the flavonoid biosynthetic pathway in** ***C. sinensis*** **leaves.**

Catechins are the main ingredient of flavonoids, which are not only important for tea quality but also related to the growth and metabolism of tea plant. The catechins pathways in the four tea plant cultivars were analyzed in this study (Figure 9). A total of 150 unigenes involved in flavonoid biosynthesis were annotated and found to encode 18 putative enzymes from integrated function annotation (COG, GO, KEGG, Swissprot, TrEMBL, Nr, and Nt annotation). These genes potentially related to catechins biosynthesis are detailed in Additional file 3.

### Catechins contents among the four tea plant cultivars detected through RP-HPLC

**Figure 10:**
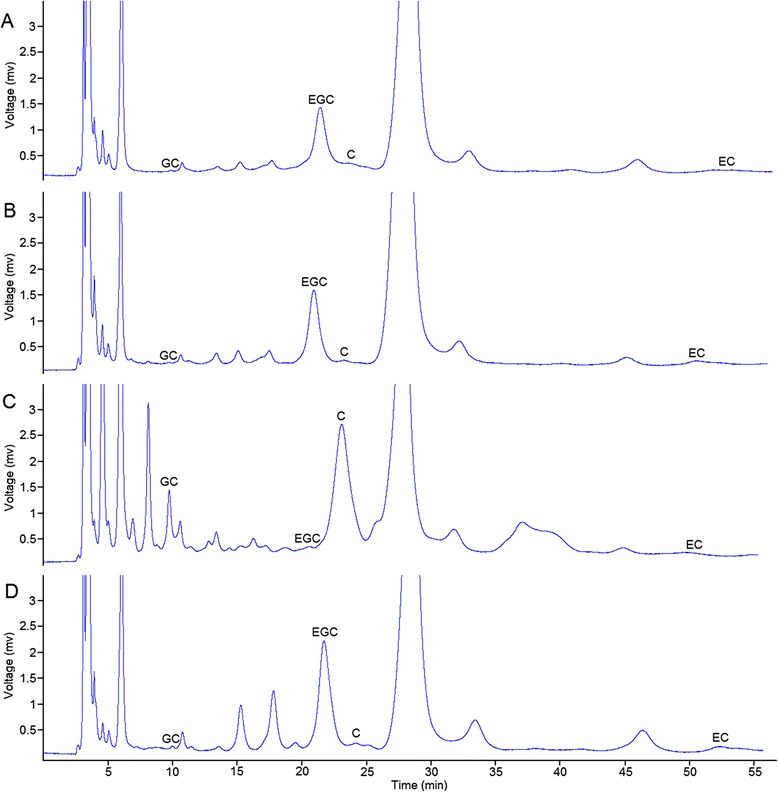
**HPLC chromatogram of catechins from four tea plant cultivars: (A)‘Yunnanshilixiang’, (B)‘Chawansanhao’, (C)‘Ruchengmaoyecha’, and (D)‘Anjibaicha’.**

**Figure 11:**
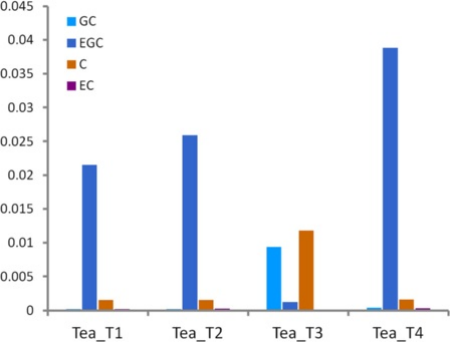
**Histogram of the GC, EGC, C, and EC contents from four tea plant cultivars.** The ordinate represents the weight of the catechins in 1 g dry tea.

RP-HPLC was performed to separate and detect catechins with high accuracy and sensitivity. The polyphenols extracted from the four parts of dry tea leaves were used for testing (Figure 10). The contents and proportions of the four components [(−)-EGC, (−)-EC, (+)-GC, and (+)-C] were highly similar in three tea plant cultivars, namely, Tea_T1, Tea_T2, and Tea_T4. The (−)-EGC and (−)-EC contents were always higher than the (+)-GC and (+)-C contents in these three tea plant cultivars. The leaves of these cultivars were similar in type and were small and medium in size. However, these detected indicators of large-leaf Tea_T3 were completely in contrast with the rest of the cultivars (Tea_T1, Tea_T2, and Tea_T4). The (−)-EGC and (−)-EC contents were obviously lower than the (+)-GC and (+)-C contents in Tea_T3 (Figure 11).

### Expression profiles of the genes involved in catechins biosynthesis in tea plant

RPKM values were used to analyze the expression of 150 unigenes involved in flavonoid biosynthesis in the four tea plant cultivars to evaluate the catechins production capacity of the tea plants (Additional file 3). The unigenes of the most and the least expression levels were taken as up-regulated and down-regulated genes, respectively. We identified 38 up-regulated and 8 down-regulated unigenes in Tea_T1, 40 up-regulated and 12 down-regulated unigenes in Tea_T2, 26 up-regulated and 64 down-regulated unigenes in Tea_T3, and 44 up-regulated and 12 down-regulated unigenes in Tea_T4. The digital expression profiles revealed a different expression pattern in Tea_T3.

**Figure 12:**
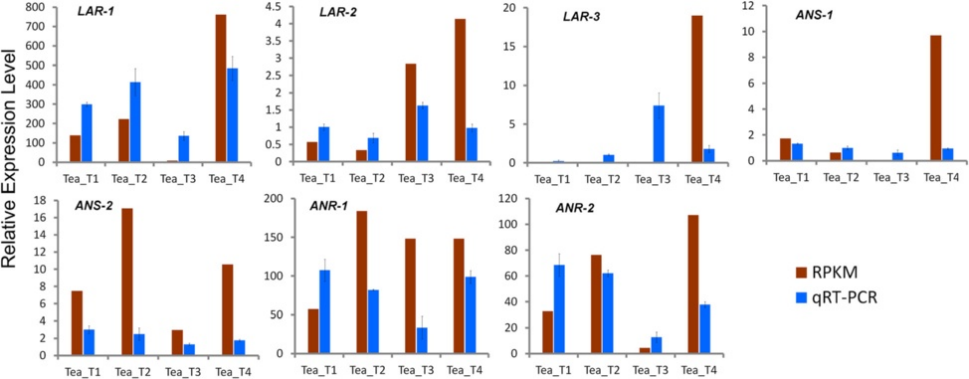
**Expression profiles of seven key genes involved in catechins biosynthesis among four tea plant cultivars.**

Seven long unigene fragments that encode for three enzymes (ANS, ANR, and LAR) at the stage of flavonoid biosynthesis were selected for verification through qRT-PCR analysis (Figure 12). The three enzymes dominated catechins production in tea plant. The results showed that the expression profiles of the seven unigenes from Tea_T3 differed from those of the unigenes from other cultivars and those five unigenes almost exactly coincided with predictable results. Moreover, six unigenes, *LAR-2*, *LAR-3*, *ANS-1*, *ANS-2*, *ANR-1*, and *ANR-2*, had strong positive correlations with corresponding catechins concentration compared with the expression profiles and the previously measured catechins contents. This result suggests that the difference in the gene expression profiles of Tea_T3 may have caused catechins diversity.

## Discussion

*C. sinensis* is a perennial cross-pollination plants that has rich genetic diversity of populations [42,43]. Phenotypic diversity of *C. sinensis* mainly embodied in plant height, flower, leaf size and locules number. Catechins are special accumulation in tea plant leaves, and their contents also have high variability in different tea plant cultivars [44]. Multi-species transcriptome sequencing provided good tool to understand the complex transcriptional regulation and metabolic pathways of different catechins components. The Illumina HiSeq™ 2000 sequencing platform was used because of its higher throughput, lower cost and greater output than Illumina Solexa Genmoe Analyzer [45-47]. Multiple samples can also be simultaneously sequenced by barcoding (multiplexing). A total of 146,342 unigenes were obtained from the tea leaf transcriptomes of the four cultivars; this number is higher than that reported from the leaves of another tea variety (25,637 unigenes) [48]. This number is between the sequencing assembly results from mixed tissues (127,094 unigenes) [29] and from cold acclimated leaves (179,753 unigenes) [49] of *C. sinensis*. The transcriptome of mixed tissues may not reach saturation by mapping it to the transcriptome of cold acclimated leaves [49]. In addition, leaves acclimated to cold temperatures express more genes than normal [49,50]. These findings indicated that the information of this study on *C. sinensis* leaves were relatively comprehensive. It is noteworthy that the data sizes of the sequences from the four samples were different. The smallest data size only had 34,442 unigenes from Tea_T3, but the largest data size had 86,523 unigenes from Tea_T1. Therefore, each of the four copies of data independently contributed to the construction of the Tea.Unigene library.

A total of 68,890 (47.07%) of the 146,342 unigenes from the Tea.Unigene library were annotated to public databases (GO, COG, KEGG, Swissprot, TrEMBL, Nr, and Nt) for comprehensive analysis. Previous studies only included 55,088 annotated unigenes from 127,094 unigenes [29], 22,872 annotated unigenes from 25,637 unigenes [48], and 53,201 annotated unigenes from 179,753 unigenes [49]. Compared with these studies, the present study obtained more complete annotation information. The annotations in this study were compared with the GO annotation from Shi et al. [29], and the principal difference was found between annotations from “response to stimulus of biological processes”, “membrane of cellular component”, and “nucleic acid binding transcription factor activity of molecular function”, this result indicated that the leaves were prominent at the molecular level in response to stimulation and membrane metabolism. The COG annotation was also compared with previous studies on *C. sinensis* [29,48]. Results showed that the unigene expression profiles under “the categories of posttranslational modification”, “protein turnover”, and “chaperones” did not specifically appear in tea leaf tissue. The present results were similar to the findings of Shi et al. [29] rather than Wu et al. [48].

The abundance of gene expression is often used to explore gene expression profiles, and DEGs are found among different developmental stages, organizations, treatments, and species [51-54]. In the present study, 217 DEGs were identified and annotated from the four tea plant cultivars. The analysis results of orthologous groups of protein from COG function classification showed that the major differences among the four tea plant cultivars were “energy production and conversion”, “posttranslational modification”, “protein turnover”, “chaperones”, “general function prediction only and secondary metabolites biosynthesis”, “transport”, and “catabolism”. However, no DEGs were found for “RNA processing and modification”, “cell cycle control”, “cell division”, “chromosome partitioning”, “nuclear structure”, “defense mechanisms”, “extracellular structures and intracellular trafficking”, “secretion”, and “vesicular transport”. Overall, the cultivars still exhibited high molecular stability.

The four tea plant cultivars (Tea_T1 to T4) from different provinces differed in leaf size, plant morphology, and stress resistance. It is generally believed that the catechins contents were higher in large-leaf species than in small-leaf species of tea plant. However, this has not been validated at the molecular level. Many genes correlate with the concentrations of catechins in the flavonoid biosynthetic pathway of *C. sinensis*. These genes include *PAL* [55], *C4H* [55], *F3H* [56], and *ANR* [57]. Almost all genes involved in catechins biosynthesis were also found in other species [58-61]. However, the expression of a particular gene does not necessarily mean a relationship with catechins contents because of the complexity of flavonoid biosynthesis and the existence of gene isomers.

Integrated functional annotation and further RPKM value analyses of the four tea plant cultivars showed that 150 unigenes were involved in the flavonoid biosynthetic pathway. Digital expression profiles revealed that different expression profile patterns may exist in Tea_T3. We further confirmed the expression variations and the relationship between the gene expression and catechins biosynthesis as follows. Three key enzyme genes (*ANS*, *ANR*, and *LAR*) were selected for qRT-PCR analysis, and four types of end products [(−)-EGC, (−)-EC, (+)-GC, and (+)-C] of the flavonoid biosynthetic pathway were selected for RP-HPLC detection. ANS to ANR are unique for the synthetic pathway of (−)-EC and (−)-EGC. The low expression levels of *ANS-1*, *ANS-2*, *ANR-1*, and *ANR-2* in Tea_T3 can explain the low contents of (−)-EC and (−)-EGC in this cultivar. LAR is involved in the synthesis of (+)-C and (+)-GC, which significantly accumulated in Tea_T3. The *LAR* gene seems to be higher expression level in Tea_T3. One of them, *LAR-1* actually had low expression in Tea_T3. These results revealed that the other two *LAR* genes (*LRA-2* and *LRA-3*) or more may control (+)-C and (+)-GC generation in tea plant.

Catechins are the largest group of secondary metabolites in tea and are very important for processing suitability and quality [36,37]. The (−)-EGCG content of catechins is the largest and next to this are (−)-EGC, (−)-ECG, (−)-EC in green tea, the (+)-C and (+)-GC contents of catechins are usually trace [62]. However, (+)-GC is considered the most important catechin for sensory quality in black tea, (+)-C is correlated positively and significantly with various individual quality attributes and total quality scores [63]. The contents and component proportions of catechins of *C. sinensis* mainly determined by the cultivars of tea plant and environmental conditions [64,65]. In the present study, four tea plant cultivars from different origins were selected for RP-HPLC detection of catechins. The results showed that the (−)-EC and (−)-EGC contents of catechins in ‘Yunnanshilixiang’ (Tea_T1), ‘Chawansanhao’ (Tea_T2), and ‘Anjibaicha’ (Tea_T4) were higher than that in ‘Ruchengmaoyecha’ (Tea_T3); the (+)-C and (+)-GC contents of catechins in Tea_T3 were higher than that in the other three tea plant cultivars. It conformed to their processing characteristics of green tea or black tea. In addition, the relevance between three key structural genes (*ANS*, *ANR*, and *LAR*) and the diversity of catechins components in the four tea plant cultivars was confirmed through analyzing their expression profiles. This will help to explore tea-processing suitability at the molecular level and develop better germplasm resources of tea plants based on the genetic metabolic regulation of catechins.

## Conclusions

This study provides a global survey of transcriptomes from four *C. sinensis* cultivars and thus may serve as an available genetic diversity resource for the tea plant. Analyses of transcriptome profiles and physiological indicators identified putative genes involved in the flavonoid biosynthetic pathway. Results showed that the multi-gene regulation of large-leafed catechins significantly differed relative to other cultivars. The expression levels of genes *ANS*, *ANR*, and *LAR* may cause differences in catechins components by comparing the expression profiles and catechins contents of the cultivars. This study provided novel insights into the mechanisms of catechins biosynthesis in tea leaves.

## Methods

### Plant material and RNA isolation

Five-year-old cutting tea plant seedlings of Tea_T1, Tea_T2, Tea_T3, and Tea_T4 were planted in a growth chamber at the Tea Science Research Institute, College of Horticulture, Nanjing Agricultural University (Nanjing, China). The plants were grown in acidic soil (pH 5.6), and the conditions were maintained at 23 ± 2°C temperature and 70 ± 10% relative humidity. Four young tea plant leaves were selected, quickly frozen in liquid nitrogen, and then stored at −80°C for RNA extraction.

RNA was extracted from the tea plants according to the instruction manual of the Quick RNA isolation Kit (Huayueyang Biotech Co., Ltd., Beijing, China). The extracted RNA was treated with RNase-free DNaseI (TaKaRa Biotech Co., Ltd., Dalian, China) to remove residual DNA. RNA integrity was checked through agarose gel electrophoresis (1.2%), and RNA concentration was estimated using an Agilent 2100 Bioanalyzer (Agilent Technologies, Inc., Santa Clara, CA, USA).

### Construction of cDNA library and illumina sequencing

High-quality RNA samples from tea plants were sent to Biomarker Technologies Corporation (Beijing, China) for cDNA libraries construction and sequencing. Magnetic oligo (dT) beads were used to enrich the poly (A) mRNA tails of four independent RNA. The enriched mRNA was fragmented into small pieces, which were prepared as templates for cDNA synthesis. Double-stranded cDNA was synthesized using SuperScript II, buffer, dNTPs, RNaseH, and DNA polymerase I. The cDNA was purified using a QiaQuick PCR extraction kit (Qiagen, Inc., Hilden, Germany) and was eluted with EB buffer. The short cDNA fragments were subjected to end repair, adapter ligation, and agarose gel electrophoresis filtration. Then, the suitable fragments were selected as templates for PCR amplification. The four constructed cDNA libraries of tea plant were sequenced using the Illumina HiSeq™ 2000 platform.

### Data filtering and *de novo* assembly

High-quality clean reads were obtained by removing the adaptor sequences, duplicated sequences, ambiguous reads (‘N’), and low-quality reads. Transcriptomes from four datasets were separately assembled *de novo* using Trinity (http://trinityrnaseq.sourceforge.net/). In brief, clean reads with a certain overlap length were initially combined to form long fragments without N. These fragments are called contigs. Related contigs were clustered using the TGICL software [66] to yield unigenes (without N) that cannot be extended on either end, and redundancies were removed to acquire non-redundant unigenes.

### Functional annotation of the assembled unigenes

The unigene sequences of the four tea plant cultivars were searched using BLASTX against the Nt, Nr, KEGG, GO, COG, Swiss-Prot, and TrEMBL databases (*E*-value ≤1E-5) to retrieve protein functional annotations based on sequence similarity. High-priority databases (followed by Nr, Swiss-Prot, and KEGG) were selected to determine the direction of the unigene sequences. The best aligning results were used to predict the coding region sequences from unigenes, and the coding sequences (CDSs) were translated into amino sequences using the standard codon table. The ESTScan software [67] was used to decide the sequence direction of the unigenes that could not be aligned to any of the above databases. GO terms were assigned to each sequence annotated by BLASTX against the Nr database using the Blast2GO program with the *E*-value threshold of 1E-5 for further functional categorization. The WEGO software [68] was used to plot the distribution of the GO functional classification of the unigenes. The unigene sequences were also aligned to the COG database to predict and classify possible functions. The unigenes were assigned to KEGG pathway annotations to analyze inner-cell metabolic pathways and the related gene function using BLASTX.

### Analysis of the functional enrichment of DEGs

The RPKM method eliminated the influence of different gene lengths and sequencing levels on the calculation of gene expression. Therefore, RPKM values were directly used to compare gene expression differences between different samples. The DESeq package was used to obtain the “base mean” value for identifying DEGs. FDR ≤0.01 and the absolute value of log2 ratio ≥1 were set as the thresholds for the significance of the gene expression difference between the two samples.

### Chromatographic conditions of RP-HPLC

The catechins contents of the four tea plant cultivars were analyzed through RP-HPLC according to GB/T8313-2008 (China). The samples were applied in the Shimadzu LC-20A series (Shimadzu Co., Kyoto, Japan). A Hedera ODS-2 C18 analytical column (250 mm × 4.6 mm i.d., 5 μm nominal particle size) was used for chromatographic separation. Gradient elution conditions were modified to better separate peaks. Double distilled water was used as mobile phase A, and primary mobile phase A was used as mobile phase B. For the gradient elution was at 2:3 mobile phase A and B ratio. The separation of the catechins was checked using a SPD-20A UV detector.

### Validation of the digital expression profiles through quantitative real-time PCR

Seven genes that encode for three enzymes (ANS, ANR, and LAR) at the late stage of flavonoid biosynthetic pathway by KEGG were chosen for validation among the four tea plant cultivars using quantitative real-time PCR. Gene-specific primers were designed using the Primer Premier 5.0 software. QRT-PCR was performed on a Bio-Rad iQ5 real-time PCR platform (Bio-Rad Laboratories, Inc., Hercules, CA, USA) using SYBR® *Premix Ex-Taq*™ (Tli RNaseH Plus), ROX plus (TaKaRa Biotech Co., Ltd., Dalian, China) according to the manufacturer’s instructions. Glyceraldehyde-3-phosphate dehydrogenase (GAPDH) was selected as the internal control gene for normalization as previously reported. The cDNAs were diluted 18-fold with nuclease-free deionized water, and 2 μL of each sample was extracted as template added to the reaction mixture (20 μL) containing 10 μL of SYBR® *Premix Ex-Taq* (2×) (Tli RNaseH Plus), ROX plus, 0.4 μL of (10 μM) each primer, and 7.2 μL of ddH_2_O. Thermal cycling was performed under the following conditions: 95°C for 30 s, 40 cycles at 95°C for 5 s, and 55°C for 20 s. Each reaction was performed in triplicate, in which the average threshold cycle was calculated to estimate the relative gene expression levels using the 2^−ΔΔCt^ method [69]. The data were expressed as the mean ± SD, and all primer information is listed in Table 4.

**Table 4:** **Primers for qRT-PCR to verify above seven genes involved in catechins biosynthesis in tea plant**

**Target CDSs**	**Primer**	**Sequence (5′ to 3′)**
Tea_T4_Unigene_BMK.28458 (*LAR-1*)	F	AAACTCTTCAAGACAAAGGCGCTAA
	R	TCTATCAATCGCCGCACCCTC
Tea_T4_Unigene_BMK.50436 (*LAR-2*)	F	GACTGTAGCAGCAGAAGTAGCC
	R	TCAATCTTATGGTCCCTCAAA
Tea_T4_Unigene_BMK.41423 (*LAR-3*)	F	GCTGTGGGTGGTGCTAAT
	R	GCGATCCAAAGGAGGAAT
Tea_T4_Unigene_BMK.49588 (*ANS-1*)	F	ATGACTACAGTGGCTGCCCCGA
	R	CAACGCCTCCCGACACCTCTC
Tea_T1_Unigene_BMK.79499 (*ANS-2*)	F	ACGAGGGCAAATGGGTCA
	R	CCTTATTAACGAGTCCACGATG
Tea_T1_Unigene_BMK.72783 (*ANR-1*)	F	CTGTCCGAGACCCAGGCAATC
	R	GGGCGTCAAAGCTCTGTTCAT
Tea_T4_Unigene_BMK.60174 (*ANR-2*)	F	CAATGGCAATGGTAACAACA
	R	TCCAGTGCTACGAGGTGAG
*TaGAPDH* (internal control gene)	F	TTGGCATCGTTGAGGGTCT
	R	CAGTGGGAACACGGAAAGC

### Availability of supporting data

The data supporting the results of this article are included within the article.

## Additional files

Additional file 1:
**List of KEGG pathway annotations in the** ***C. sinensis*** **transcriptome.**
Additional file 2:
**List of annotation information of differentially expressed genes among four tea plant cultivars transcriptomes.**
Additional file 3:
**Unigenes involved in the flavonoid biosynthesis in leaves of** ***C. sinensis.***

## Abbreviations

RPKM: Reads per kb per million reads
Nt: Non-redundant nucleotide database
Nr: Non-redundant protein database
Swiss-Prot: Annotated protein sequence database
TrEMBL: Computer-annotated supplement to Swiss-Prot database
KEGG: Kyoto encyclopedia of genes and genomes
GO: Gene ontology
COG: Clusters of orthologous groups of protein
DEGs: Differentially expressed genes
RP-HPLC: Reversed-phase high-performance liquid chromatography
qRT-PCR: Quantitative real-time PCR
GAPDH: Glyceraldehyde-3-phosphate dehydrogenase
